# Cytokine and Chemokine Recovery Is Increased by Colloid Perfusates during Dermal Microdialysis

**DOI:** 10.3390/ma11050682

**Published:** 2018-04-27

**Authors:** Sven R. Quist, Claudia Kirbs, Charlotte Kloft, Harald P. Gollnick

**Affiliations:** 1 Department of Dermatology and Venereology, Otto-von-Guericke University, D-39120 Magdeburg, Germany; harald.gollnick@med.ovgu.de; 2 Dermatology Centre MDZ Mainz, D-55128 Mainz, Germany; 3 Department of Clinical Pharmacy, Institute of Pharmacy, Martin-Luther-Universitaet Halle-Wittenberg, D-06120 Halle, Germany; claudia.kirbs@fu-berlin.de (Cl.K.); charlotte.kloft@fu-berlin.de (Ch.K.); 4 Department of Clinical Pharmacy and Biochemistry, Institute of Pharmacy, Freie Universitaet Berlin, D-14195 Berlin, Germany

**Keywords:** cytokines, colloid perfusates, microdialysis, skin, recovery

## Abstract

Cytokines and chemokines play important roles in cell signalling, and microdialysis is a promising tool for monitoring these inflammation markers ex vivo. Therefore, the collecting of these mediators at the highest concentrations possible is crucial. Depending on the size of the mediator of interest, the collection of these high molecular mass molecules has thus far been difficult due to their low recovery, even when using high cut-off (100 kDa) microdialysis membranes. This study aimed to optimize the recovery of various cytokines and chemokines by validating the use of different perfusates in cutaneous microdialysis, and comparing intravenous (i.v.) colloids, crystalloids, and a lipid emulsion formulations that are approved for i.v. applications. Methods: In vitro and in vivo recovery experiments using six recombinant cytokines varying in molecular size (interleukin-2 (15 kDa), interleukin-6 (20.5 kDa), interleukin-8 (8 kDa), interleukin-12p70 (70 kDa), TNF-α (17.5 kDa), and vascular endothelial growth factor (VEGF) (38 kDa)) were performed in the presence of different perfusates for i.v. applications: Ringer’s lactate, dextran 60 kDa, hydroxyethyl starch 70 kDa, and hydroxyethyl starch 200 kDa solutions as well as a lipid emulsion formulation. Recovery was determined through (i) microdialysis of cytokines and chemokines in Ringer’s lactate solution or human serum in vitro, and (ii) retrodialysis of excised porcine and human skin cadavers in vitro and porcine skin in vivo. Furthermore, we used skin trauma (catheter insertion) and Ultraviolet B irradiation of 3 × 3 cm^2^ skin areas to sample cytokines and chemokines in vivo and compared the amounts that were obtained using crystalloid and colloid perfusates. All the cytokines and chemokines within the dialysates were quantified through a flow cytometry-based bead array assay. Results: Overall, recovery was strongly increased by the colloids, particularly hydroxyethyl starch 70 kDa, in vitro, ex vivo, and in vivo. When compared with the recovery achieved using Ringer’s lactate, this increase was most effective for proteins ranging from 8 to 20.5 kDa. Hydroxyethyl starch 70 kDa significantly increased the recovery of interleukin (IL)-8 in human serum in vitro when compared with Ringer’s lactate. More cytokines and chemokines were recovered using colloids compared with crystalloids. However, the increase in recovery values was lower for IL-12p70 and VEGF. Conclusions: Regarding the dialysate volumes and final dialysate concentrations, colloid perfusates are overall superior to crystalloid perfusates, such as Ringer’s lactate, when sampling cytokines and chemokines, resulting in higher recoveries. However, the sampling of high-molecular-mass cytokines during microdialysis remains challenging, and experimental in vitro data are not completely comparable with data obtained ex vivo or in vivo.

## 1. Introduction

**Figure 1:**
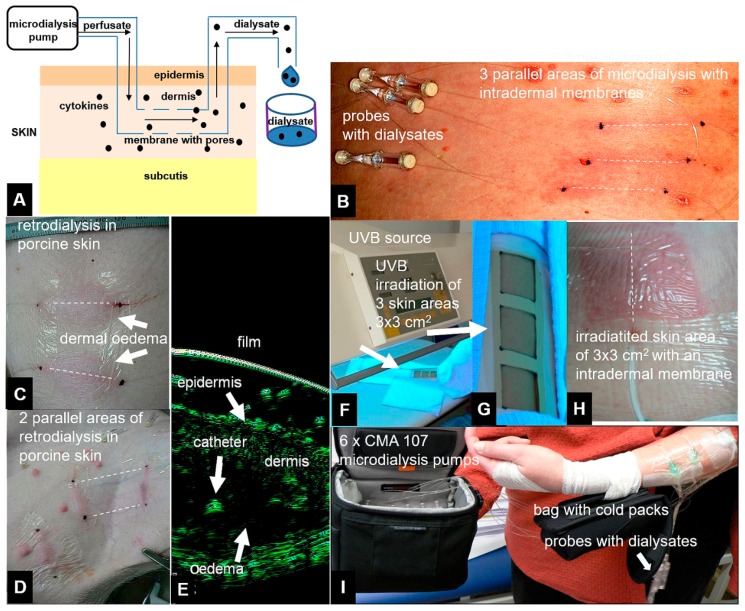
Principle of the microdialysis experiments: 100 kDa cut-off membranes perfused with crystalloid or colloid solutions (perfusates) were placed in the dermal skin using a microdialysis pump at flow rates of 0.2, 0.5, or 1 µL/min. The dialysates were collected in fractions after an exchange process within dermal skin between the interstitial fluid and the perfusate via a microdialysis membrane (scheme, **A**). Clinical pictures of linear microdialysis cut-off membranes (CMA) 66 catheters placed in human skin (**B**). Retrodialysis of porcine skin indicated cytokine release from the catheter to the dermis. Signs of inflammation in the catheter areas included rubor and tumefaction (**C**,**D**) as well as dermal oedema surrounding the microdialysis catheter (**E**), as demonstrated by high-resolution 22 MHz ultrasound (Taberna Pro Medicum, Luneburg, Germany). After placement of the microdialysis catheters into the dermal skin of a human forearm, UV irradiation and skin erythema were used to generate cytokines and chemokines (**F**–**H**). Dialysates were collected for 48 h after UVB irradiation in this clinical experiment (**I)**. Dotted lines indicate virtual intradermal placement of microdialysis membranes.

Dermal microdialysis (DM) is a method currently used to monitor tissue chemistry in vivo, including tissue physiology, pathophysiology, drug efficacy, and drug tissue concentrations, and is feasibly applicable to almost every human organ, including skin [1,2,3,4,5,6]. The method consists of placing a very thin, semipermeable, hollow catheter that was made of polyarylethersulphone (PAES) in the dermis or subcutis. This catheter functions as an artificial vessel and is used to perfuse the tissue with a tissue-compatible sterile buffer at a low flow rate by means of a microdialysis pump (Figure 1A) [1,2,7,8]. The introduction of 100 kDa cut-off membranes, which have now been approved for clinical use in humans, has increased the range of feasibly detected analytes, including inflammatory mediators, such as cytokines and chemokines (Figure 1B) [9,10,11,12]. Cytokines and chemokines play important roles in cell signalling in skin inflammation, such as psoriasis [13,14]. Previous studies have shown that cytokines and chemokines can be monitored in dermal and subcutaneous skin as they are released following a stimulus, such as Ultraviolet B irradiation, heat, or pain [9,10,11]. However, conventional sampling of high-molecular-mass proteins (>10 kDa) remains challenging due to their low recovery, even when higher cut-off membranes of 100 kDa are used [15,16,17,18]. Multiplex immunoassay technology has recently become available for simultaneously measuring panels of various cytokines and chemokines in sample volumes that are as low as 50 µL [19,20,21,22]. Multiplex arrays have already been established to quantify cytokines in dermal and subcutaneous skin [9,11], allowing for more flexibility and quantification in small sample volumes [19,21,22]. Low recovery of macromolecules, such as high-molecular-mass cytokines and chemokines, is influenced by different levels of colloid osmotic pressure on both sides of a semipermeable membrane. In cases of crystalloid perfusates, such as 0.9% NaCl or Ringer’s lactate solutions, the colloid osmotic pressure outside the microdialysis membrane in the tissue of interest prevents the diffusion of macromolecules into the catheter lumen. Furthermore, even if macromolecules are capable of passing through the membrane, water, or other small soluble molecules will pass through the membrane in both directions much more easily and faster than macromolecules, which is known as ultrafiltration [23,24,25]. Since ultrafiltration can result in perfusate loss and low macromolecule recovery, the use of colloid perfusates with water-binding and colloid osmotic pressure capacity can increase the recovery of high-molecular-mass cytokines and chemokines [26]. Colloid solutions, such as dextrans, gelatine derivatives, hydroxy ethyl starches, and human albumin are large, oncotically active molecules that are used intravenously (i.v.) as volume plasma expanders in critical care medicine to retain water within the plasma space in patients with hypovolemic shock. Dextran solutions are currently used as a perfusate to avoid ultrafiltration and to sample macromolecules from microdialysis in muscle and adipose tissues in vivo. However, these are associated with a high risk of anaphylaxis or allergic reaction [27,28,29]. Therefore, we tested other i.v. solutions that were approved for clinical use as potential perfusates that might be at least similar to or even more effective than dextran 60 kDa solution with respect to their recovery and use within dermal skin to sample cytokines and chemokines in vitro and in vivo. We analysed the recoveries of different crystalloids, dextran 60 kDa and other perfusates, including hydroxyethyl starch (HES) solutions of 70 kDa and 200 kDa and a lipid emulsion formulation (soy bean phospholipids and triglycerides) used i.v. or as a source of parenteral nutrition. The term recovery reflects the relationship between the concentration of a compound in the periprobe fluid and in the dialysate. Recovery can be determined either as the absolute recovery (AR), which is defined as the total amount extracted by the dialysate during a time period, or the relative recovery (RR), which reflects the concentration of a compound in the dialysate divided by the concentration in the periprobe fluid [30,31,32]. In vivo, the real concentration of a compound is mostly unknown. Therefore, a reverse dialysis, termed retrodialysis, is mainly used to determine the in vivo recovery [30,32,33,34]. This method is easy, useful, and applicable in any tissue of interest and has frequently been used to determine recovery in tissues. In retrodialysis, the compound is added to the perfusate, and then the in vivo loss is measured based on the calculation of its loss from the perfusate during retrodialysis [35]. The concentration equilibrates to a conventional microdialysis state based on the assumption that the concentrations inside and outside the microdialysis membrane do not differ in an equilibrate state [30,31,32]. 

In our study, we determined the RR and AR from in vitro experiments as well as relative loss (RL, cytokine concentration) and absolute loss (AL, total amount of cytokine that is lost from the perfusate) from ex vivo and in vivo experiments using retrodialysis.

To investigate the effects of different perfusates on macromolecule recovery, we first tested cytokines ranging from 8 to 70 kDa mixed together in one solution (interleukin (IL)-2 (15 kDa), IL-6 (20.5 kDa), IL-8 (8 kDa), IL-12p70 (70 kDa), tumour necrosis factor (TNF)-α (17.5 kDa), and vascular endothelial growth factor (VEGF) (38 kDa)). Recovery was investigated using an in vitro model, and conventional microdialysis was also performed. The membrane and catheter were placed in syringes that were containing either Ringer’s lactate or human serum, which was chosen to mimic the interstitial fluid that surrounds the membrane when placed in living tissue. Although not identical, human serum is quite similar to interstitial fluid [36,37]. Furthermore, for the in vivo analysis, we subjected human and porcine skin cadavers and porcine dermal skin from anaesthetized domestic pigs to retrodialysis (i.e., inverse microdialysis). As performing retrodialysis within living human skin and perfusing skin with the high doses of cytokines and chemokines that are necessary to determine their recovery would be unethical, we used two stimuli, (i) skin trauma, and (ii) UVB irradiation, to generate cytokines and chemokines within the dermal skin of the human forearm. We then performed microdialysis using crystalloid or colloid perfusates to sample cytokines and chemokines in vivo that are known to be induced by either skin trauma or UVB irradiation within dermal skin: IL-2, IL-6, IL-8 and TNF-α as in the in vitro and ex vivo experiments as well as IL-10, interferon gamma-induced protein (IP)-10, and monocyte attractant protein (MCP)-1 [9,10,11].

## 2. Methods

### 2.1. Microdialysis

For all of the in vitro experiments as well as the ex vivo and in vivo retrodialysis experiments, CMA 66/30 linear 100 kDa cut-off microdialysis catheters, with PAES membranes (30 mm in length and 0.5 mm in diameter), a 400-mm polyurethane inlet tube and a 100 mm outlet tube (both with diameters of 0.38 mm) (CMA Microdialysis, Solna, Sweden), were used. Three-millilitre syringes (CMA 106 syringes, CMA Microdialysis) were filled with the perfusate using a portable, battery-driven, and flow rate-adjustable microdialysis pump at rates of 0.1, 0.2, 0.5, 1, and 5 µL/min (CMA 107, CMA Microdialysis). For all four perfusates (Ringer’s lactate, HES 70, HES 200, and dextran 60), flow rates of 1, 0.5, and 0.2 µL/min were investigated. After performing basal microdialysis to equilibrate each flow rate for 30 min, microdialysis was performed to obtain a total volume of 50 µL, the amount needed for further analysis via the cytometric bead array assay, as follows: 50 min at 1 µL/min, 100 min at 0.5 µL/min, and 250 min at 0.2 µL/min. The results were obtained from three independent experiments with three separate membranes for the in vitro and ex vivo experiments and from two individuals for the in vivo experiments in living animals and humans.

### 2.2. Perfusates

The following perfusates, all being suitable for i.v. administration and used in critical care medicine, were investigated: (i) colloids consisting of varying molecular mass HES solutions of 70 kDa (HES 70/0.5, HAES-Rheopond 70^®^ 6% solution, Serag-Wiessner AG, Naila, Germany) and 200 kDa (HES 200/0.5, freeflex-HAES sterile 6% solution, Fresenius Kabi, Bad Homburg, Germany) with a substitution pattern (C2:C6) of 0.5 and a 6% dextran 60 kDa solution (dextran 60, Deltadex 60 6% solution, Deltaselect, Pfullingen, Germany); (ii) the crystalloid solution Ringer’s lactate (Berlin Chemie AG, Berlin, Germany); and, (iii) the lipid emulsion Lipvenös 30 (Fresenius Kabi, Bad Homburg, Germany) comprising 30% soy bean oil, 2% glycerol, and 1.2% egg phospholipids (75–81% 3-sn-phosphatidylcholine), with a known osmolarity of 219 mosmol/L, density of 0.98 g/cm^3^, and dynamic viscosity of 3.5 mPa∙s at 25 °C, as indicated by the manufacturer. The kinematic viscosities of the crystalloid and colloid perfusates tested and fluids used in this study were determined using an Ubbelohde viscometer (Schott, Mainz, Germany) at 28 °C, 32.5 °C, 34.5 °C, and 37 °C, and the dynamic viscosities were calculated from these results. The pycnometric densities, kinematic and dynamic viscosities at 28 °C, and osmolarities of all of the perfusates investigated herein are listed in Table A1, and these was no significant variability between the different conditions investigated.

### 2.3. Cytokines

Six recombinant, lyophilized exclusive human cytokines and chemokines of increasing molecular mass, but below the catheter’s 100 kDa cut-off with importance in skin inflammation were used in the initial solution for the in vitro, ex vivo and in vivo experiments in anaesthetized pigs (molecular masses, as indicated by the supplier): IL-2 (15 kDa), IL-6 (20.5 kDa), IL-8 (8 kDa), IL-12p70 (70 kDa), TNF-α (17.5 kDa), and VEGF (38 kDa) (all from BD Bioscience, Heidelberg Germany). Stock solutions with concentrations of 10 ng/mL were generated at on the day of the experiment and were further diluted. For the human in vivo experiments using UVB irradiation or trauma, the following cytokines, which are known to be induced by either skin trauma or UVB irradiation within dermal skin, as reported previously, were analysed: IL-2, IL-6, IL-8, TNF-α (the same cytokines analysed in the previous experiments), IL-10 (18 kDa), IP-10 (10 kDa), and MCP-1 (8.7 kDa).

### 2.4. In Vitro Cytokine Recovery from Ringer Lactate Solution and Human Serum

To determine the cytokine RRs and ARs in vitro, all six recombinant cytokines were diluted from the stock solutions to 1600 pg/mL with Ringer’s lactate or human serum, filled into 1-mL syringes (BD Bioscience) as the initial cytokine solution, and were placed on a shaker. The in vitro recoveries of the six mixed human cytokines and chemokines (IL-2, IL-6, IL-8, IL-12p70, TNF-α and VEGF) in two different solutions were investigated at flow rates of 1, 0.5, and 0.2 µL/min, as described above. First, the crystalloid Ringer’s lactate solution was used to determine the basal cytokine recovery levels in vitro, i.e., as one of the perfusates and in the medium surrounding the membrane (first in vitro experiment). To mimic human interstitial fluid outside the membrane, human serum was used in the second in vitro experiment and filled into syringes, as described above. Human serum was isolated from the venous blood of a 38-year-old volunteer by centrifugation at 3500× *g* for 5 min. He gave informed consent, and the blood was collected in accordance with the recommendations of the local review board (approval 81/07), German Medical Council and the Declaration of Helsinki. The serum contained 35 g/L total protein with an osmolarity of 300 mosmol/L, similar to previous reports [38,39]. In vitro recovery of the six mixed human cytokines and chemokines (IL-2, IL-6, IL-8, IL-12p70, TNF-α, and VEGF) at microdialysis flow rates of 1, 0.5, and 0.2 µL/min was carried out as previously described. The RR was calculated as follows: RR (%) = CD·100/CICS (CD: concentration of the dialysate in pg/mL; CICS: concentration of the initial cytokine solution in pg/mL). The AR incorporated any differences in the dialysate sample volume from the expected 50 µL and was calculated, as follows: AR (%) = CD·(final dialysate sample volume in µL/50)·100/CICS.

### 2.5. Ex Vivo Cytokine and Chemokine Recovery from Human and Porcine Cadaver Skin Using Retrodialysis

To assess ex vivo recovery from a tissue environment that more closely represents the in vivo situation when performing DM and to study the possible influence of any tissue adherent effects on recovery, we used the retrodialysis method, which is basically inverse microdialysis, on excised human and porcine cadaver skin. Cadaver tissue has been previously used to study dermopharmacy, tissue physiology, and biochemistry [40,41,42,43]. For this study, full skin samples (3 × 2 cm^2^) from human volunteers (harvested after receiving informed consent and collected according to recommendations from the German Medical Council and local review board as well as from the Declaration of Helsinki) and domestic pigs (10 × 20 cm^2^) containing the epidermis, dermis, and subcutis were collected. The skin samples were fixed on plastic culture dishes with 21G needles and were moisturized with 1 mL of Ringer’s lactate solution around the subcutis every 30 min to prevent the tissue from drying out. For recovery experiments using retrodialysis, all six recombinant human-specific cytokines were diluted to 1600 pg/mL in each perfusate andwere filled into 3 mL CMA 106 syringes (CMA Microdialysis) to obtain the initial cytokine solution for retrodialysis. Next, 100 kDa cut-off CMA 66 microdialysis membranes were placed in the dermis, controlled by a high-resolution 22 MHz ultrasound (Taberna Pro Medicum, Lüneburg, Germany). Retrodialysis was performed using the same flow rates and dialysis periods described previously. The dialysate volumes and final cytokine concentrations were analysed, as described above. The RLs of the retrodialysis experiments were calculated as follows: RL (%) = 100 − [CD·100/CICS] (CD: concentration of the dialysate in pg/mL; CICS: concentration of the initial cytokine solution for retrodialysis in pg/mL). ALs were calculated as AL (%) = 100 − [CD·(final dialysate sample volume in µL/50)·100/CICS)].

### 2.6. In Vivo Cytokine Recovery from Anaesthetized Porcine Skin Using Retrodialysis

Because of its similarity to human skin with respect to permeability, density, hair follicle diameter, and epidermal and dermal skin structures (thickness, lipids, elastic fibres), porcine skin is a good in vivo model for studying recovery with microdialysis [3,5,17].

For in vivo cytokine recovery in porcine skin, DM was performed in a sedated, intubated, and isoflurane-anaesthetized domestic pig (Figure 1C,D) for up to 7 h using the retrodialysis method (reviewed by the review board for animal health). Up to ten microdialysis catheters (100 kDa cut-off CMA 66/30 membranes, CMA Microdialysis) were placed in the dermal porcine skin at least 6 cm apart, and catheter placement was controlled by high-resolution ultrasound (Figure 1E). The microdialysis catheters were placed at a depth of 0.9–1.2 mm and were controlled by a 22 MHz ultrasound. The microdialysis catheters were perfused with mixed cytokine standards (1600 pg/mL IL-2, IL-6, IL-8, IL-12p70, TNF-α, and VEGF (all from BD Biosciences, Cytometric Bead Array (CBA) Flex Sets)) in dextran 60 (Deltadex 60) at 0.2, 0.5, or 1 μL/min. The RL was calculated as the difference between the starting concentration and the concentration found in the dialysate, which was the percentage of mediator that passed through the membrane into the surrounding dermal tissue in accordance with the retrodialysis calculation. Retrodialysis of the final cytokine solution led to the inflammatory skin responses rubor, tumefaction, and oedema around the catheter (Figure 1C–E). Flow rates and microdialysis pumps were used, and dialysate analyses and AL and RL calculations for retrodialysis were performed, as described above, for the ex vivo experiments. In contrast to the retrodialysis experiments, a standard RR was determined directly to compare retrodialysis with conventional microdialysis. For this purpose, a solution of the six recombinant cytokines diluted to 1600 pg/mL in Ringer’s lactate solution was injected intradermally into a defined area of 10 cm^2^ surrounding the microdialysis membrane. Microdialysis was performed at 1 µL/min using the three different perfusates (HES 200/0.5, HES 70/0.5 and dextran 60 solution).

### 2.7. In Vivo Cytokine and Chemokine Collection after Skin Trauma (Insertion) and UV Irradiation

Both retrodialyzed cadaver and porcine skin are ex vivo or in vivo models that closely represent the human situation in vivo, but they do not completely reproduce it. Whereas, in vitro experiments lack the influence of dermal tissue surrounding the catheter and a continuous fluid exchange by tissue vessels, cadaver tissue also lacks the exchange between blood and lymphatic vessels that occurs in living organisms. Therefore, we performed an in vivo experiment using living human dermal skin. However, because perfusing living human skin with a relatively high dose of a pro-inflammatory cytokine mix (1600 pg/mL) results in redness and pain, and was thus regarded as unethical by the local review board, we used a different approach. As previously reported [10,11], we induced skin trauma by inserting a catheter into human dermal skin and irradiated these skin areas with UVB light. Briefly, after receiving informed consent and approval from the local review board, comprising medical faculty from Otto von Guericke University in Magdeburg, Germany, two healthy volunteers that were aged 26–28 years with no UVB exposure or signs of skin inflammation within the test region within the last six weeks were included in this clinical experiment (approval 81/07), and the principles of the Declaration of Helsinki were followed. The minimal erythaematous dose (MED) for each volunteer was first determined by the administration of five increasing doses of UVB (Waldmann UV 3003K, Herbert Waldmann GmbH & Co. KG, Villingen-Schwenningen, Germany) to the contralateral forearm, ranging from 50 to 250 mJ/cm^2^. Three microdialysis catheters and the same 100 kDa cut-off membranes (CMA Microdialysis, Sweden) that were utilized for the collection of cytokines and chemokines were used, as previously described, and placed into the dermis on the volunteer’s volar forearm at a depth of 0.7–1.2 mm with the aid of 22 MHz ultrasound. Placement of a microdialysis catheter is known to induce an inflammatory response that is clearly detectable for up to 10–16 h post-placement [44]. Therefore, UVB irradiation was performed precisely 16 h after catheter placement (first stimulation: trauma). Skin areas of 3 × 3 cm^2^ above the dermal catheters were irradiated with 2× MED (450 mJ/cm^2^) of UVB (Figure 1F–H). The membranes were first flushed with equilibration and basal microdialysis buffers at 5 µL/min for 2 h to eliminate the initial mediators that were induced by catheter insertion. The membranes were then perfused with either Ringer’s lactate, HES 200/0.5 or dextran 60 solution, all at the same flow rate of 0.2 µL/min, for cytokine collection using the same mobile CMA107 microdialysis pump (CMA Microdialysis), which allowed free mobility throughout the experiment (Figure 1I). Microdialysate samples were collected 2 h after insertion of the catheter and then at 4 h intervals up to 62 h and immediately stored in liquid nitrogen. Like in the previous experiments, the cytokine contents in the microdialysates were quantified together using a cytokine multiplex array [20] and the CBA Flex Set for the FACSCanto II instrument (BD Bioscience). The levels of the cytokines IL-2, IL-6, IL-8, IL-10, and TNF-α and the chemokines IP-10 and MCP-1, which are known to be induced by either skin trauma or UVB irradiation, were measured at a detection limit of 2.5 pg/mL. However, IL-12p70 and VEGF were not analysed in this experiment, because these mediators are not adequately induced by the external stimuli reported previously. Furthermore, based on the local review board recommendation, we compared only three perfusates, selecting Ringer’s lactate, dextran 60, and HES 200/0.5 for this experiment (HES 70/0.5 was excluded and was not regarded as the first priority compared with HES 200/0.5 when the experiments were performed). From the data obtained, an area under the curve (AUC) was calculated using MedCal^®^ (MedCal, Ostende, Belgium), and the cytokine levels that were detected by the FACSCanto II instrument were quantified with FCAP Array v1.0.1 (BD Biosciences), as reported previously [10,20].

### 2.8. Cytometric Bead Array 

Cytometric bead arrays (CBAs, BD Bioscience, Heidelberg, Germany) allow for the simultaneous measurement of up to 30 species-specific (in our study only human-specific) mixed cytokines in sample volumes as low as 50 µL [20,21]. The results obtained using cytometric-based multiplex bead arrays are comparable to ELISA results [20,45], and CBAs have been validated on dual-laser flow cytometry platforms, such as the FACSCanto [46]. 

For standard curves, lyophilized, human-specific, standardized recombinant cytokines were diluted to 5000 pg/mL with BD assay diluents (BD Bioscience), and were subsequently further diluted 1:1 to 5 pg/mL. For each concentration on the standard curve, 50 µL of the dialysates obtained from the recovery experiments or the initial cytokine solution were incubated with a mixture comprising six human cytokine-specific capture beads (BD Bioscience) for 1 h at room temperature. This assay was followed by incubation with a mixture of six human cytokine-specific detection reagents (BD Bioscience) for 2 h at room temperature in the dark. The beads were washed with wash buffer (BD Bioscience) and centrifuged at 200× *g* for 5 min. The bead pellets were resuspended in wash buffer and were measured with a FACSCanto dual-laser flow cytometer (BD Bioscience) at 488 nm and 633 nm.

### 2.9. Sample Concentrations Calculations 

To determine the cytokine concentrations in the samples, cytokine-specific bead populations were further analysed with FCAP Array software version 1.0.1 (BD Bioscience, Heidelberg, Germany). The standard curve coefficients of determination (r^2^), as related to a five-parameter logistics equation (y = D + ((A − D)/(1 + [X − E/C]B)), were ≥0.9995 for each cytokine. The dialysate volumes were calculated by weight and density, and the results were used to determine the AR and AL.

### 2.10. Statistical Analysis

For the statistical analysis, dialysate volumes and final recoveries were calculated and presented as the means from three independent in vitro and ex vivo experiments, together with the standard deviation that was calculated using Microsoft EXCEL (Office 2007, Unterschleißheim, Germany). Furthermore, Student’s *t*-test was used to evaluate for significance differences using MedCal^®^ (MedCal, Ostende, Belgium). The results from the porcine and living human skin in vivo experiments are based on two individuals (pigs and humans, *n* = 2, presented as pooled data).

## 3. Results

### 3.1. In Vitro Cytokine and Chemokine Recovery from Ringer’s Lactate

We first analysed the RR and AR from Ringer’s lactate, which is a surrounding solution without any macromolecules or oncotic pressure, in an in vitro model where the catheter was surrounded by fluid retained in a syringe using different perfusates. Microdialysis of the six mixed cytokines using Ringer’s lactate as the surrounding medium in vitro initially showed a significant decrease in the volume obtained with the crystalloid perfusate Ringer’s lactate, particularly at low flow rates of 0.2 and 0.5 µL/min, but a strong increase in all of the colloid perfusates over the expected volume of 50 µL was observed (Table 1). Colloid perfusates increased the dialysate volume at 0.2 µL/min by ~4 fold and at 0.5 and 1 µL/min by ~2 fold (Table 1). The ARs from Ringer’s lactate solution showed an overall significant increase, especially for the 8 kDa IL-8 (Figure 1A–D), when using colloid perfusates, such as dextran 60 HES 200/0.5 and HES 70/0.5. The in vitro ARs of cytokines and chemokines from Ringer’s lactate solution were negatively correlated with the molecular mass and flow rate, resulting in high ARs for the low-molecular-mass chemokine IL-8 and the cytokines IL-2, IL-6, and TNF-α at 0.2 µL/min. In contrast to the 35 kDa VEGF, where the AR was increased up to 113% with HES 70/0.5, the AR increase in the high-molecular-mass cytokine IL-12p70 (70 kDa) was lower and only modestly increased to 7% by the same colloid perfusate HES 70/0.5 (Figure 1A–D). In contrast, the RR (concentration recovery) for IL-8 was significantly higher when the crystalloid Ringer’s lactate was used as the perfusate in contrast to the colloid perfusates at 0.2 µL/min and 0.5 µL/min (Figure 1A).

### 3.2. In Vitro Cytokine and Chemokine Recovery from Human Serum

**Figure 2:**
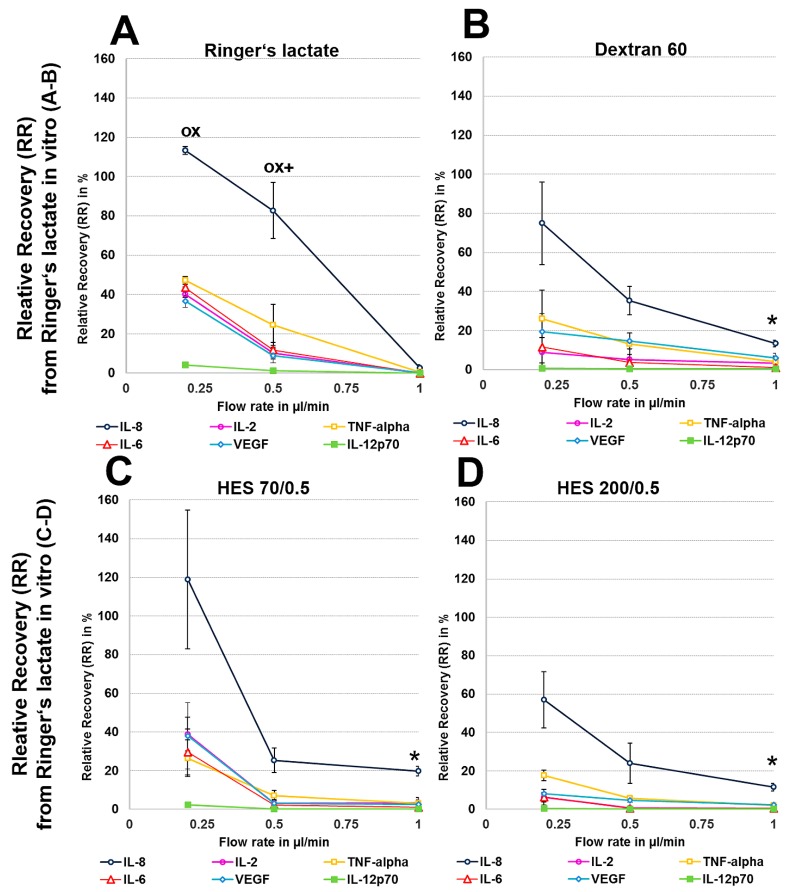

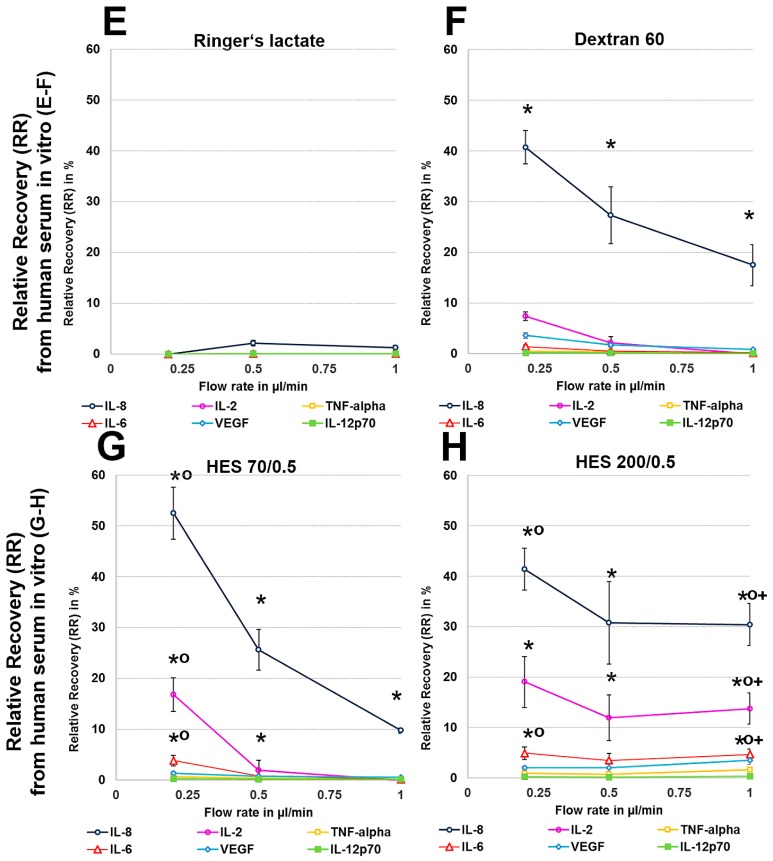
(**A**–**H**): Effect of different colloid perfusates (dextran 60 (**B**,**F**), hydroxyethyl starches (HES) 70/0.5 (**C**,**G**) and 200/0.5 (**D**,**H**) and the crystalloid Ringer’s lactate solution (**A**,**E**)) on relative recovery (RR) in Ringer’s lactate solution (**A**–**D**) and human serum (**E**–**H**) Each figure represents the mean values with standard deviation of three independent experiments of all six cytokines at flow rates of 0.2, 0.5 and 1 µL/min. Significance (*p* < 0.05) is indicated by *, x, o and +. * shows significant differences compared with Ringer’s lactate, x shows significant differences when compared with HES 200/0.5, + shows significant differences compared with HES 70/0.5, and o shows significant differences as compared with dextran 60 solution.

**Figure 3:**
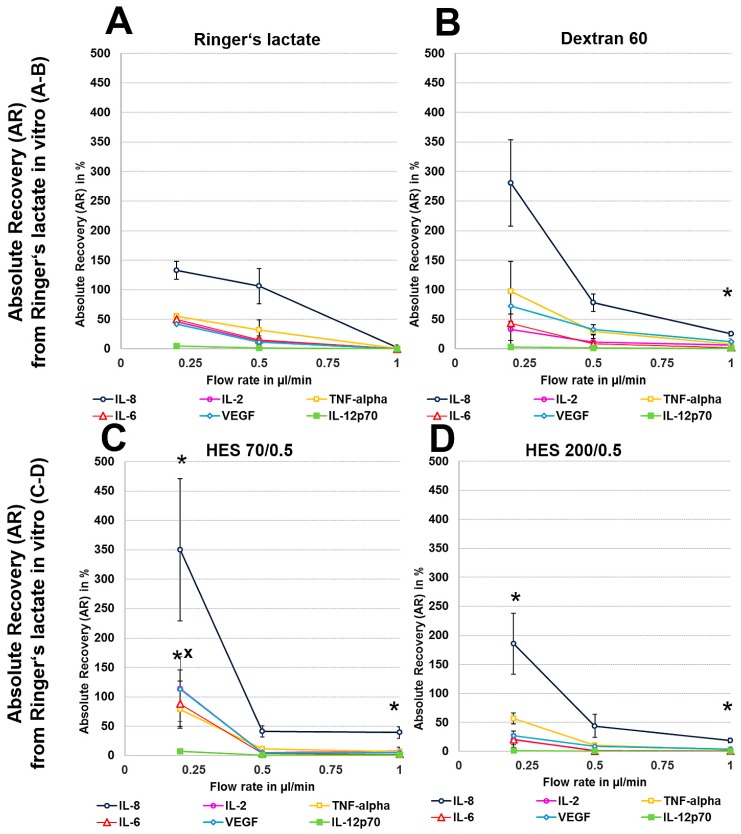

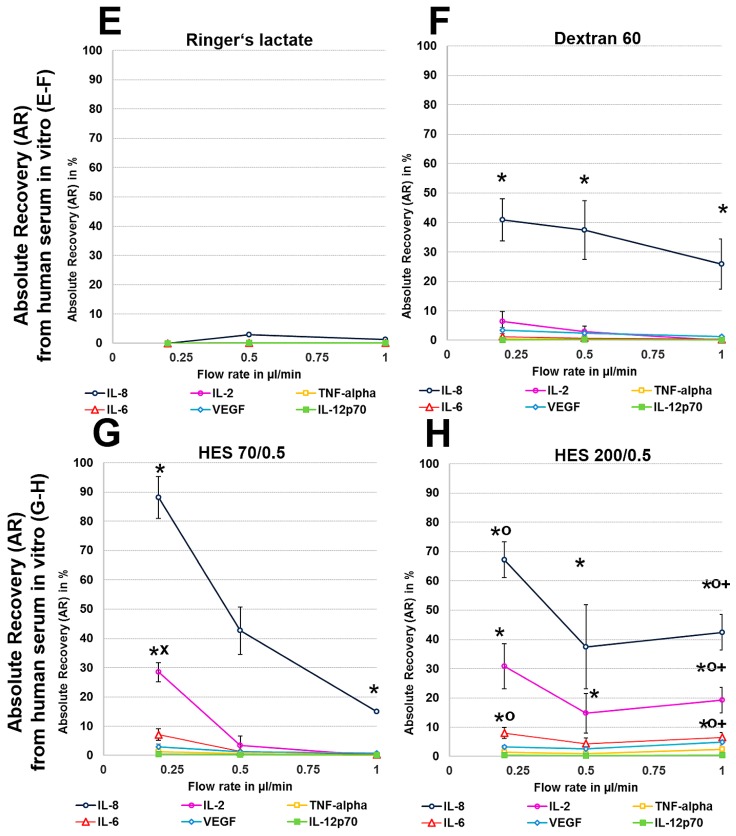
(**A**–**H**): Effect of different colloid perfusates (dextran 60 (**B**,**F**), hydroxyethyl starches (HES) 70/0.5 (**C**,**G**) and 200/0.5 (**D**,**H**) and the crystalloid Ringer’s lactate solution (**A**,**E**)) on absolute recovery (AR) in Ringer’s lactate solution (**A**–**D**) and human serum (**E**–**H**) in vitro. Each figure represents the mean values with standard deviation of three independent experiments of all six cytokines at flow rates of 0.2, 0.5 and 1 µL/min. Significance (*p* < 0.05) is indicated by *, x, o and +. * shows significant differences compared with Ringer’s lactate, x shows significant differences compared with HES 200/0.5, + shows significant differences compared with HES 70/0.5, and o shows significant differences compared with dextran 60 solution.

Human serum, with a 35 g/L protein concentration and higher osmolarity, protein level and macromolecule content than the Ringer’s lactate solution (300 vs. 279 mosmol/L, for the latter see Table A1), had a decreasing effect on dialysate volume at 0.2 µL/min because no dialysate was detectable with the crystalloid Ringer’s lactate as the perfusate (Table 1). In contrast, with colloid solutions, especially HES 70/0.5, this volume ranged from 77 to 88 µL at different flow rates, but these dialysate volumes obtained from human serum in vitro were significantly lower than those that were obtained from Ringer’s lactate (Table 1). Whereas, Ringer’s lactate has a lower osmolarity than serum, the osmolarities of colloid solutions are higher than those of serum (Table A1). The ARs and RRs of all cytokines, even IL-8, were very low using the crystalloid Ringer’s lactate as the perfusate. Colloid solutions, especially HES solutions 70/0.5 and 200/0.5, significantly increased the ARs and RRs and dialysate concentrations of the lower-molecular-mass cytokines IL-2, IL-6, and TNF-α (15–20.5 kDa), and the 8-kDa chemokine IL-8 (Figure 2 and Figure 3). In contrast to HES solution 70/0.5, HES solution 200/0.5 exhibited similar AR and RR values at flow rates between 0.5 and 1 µL/min. Overall, similar to the in vitro experiments using Ringer’s lactate instead of human serum as the surrounding medium, the cytokine, and chemokine ARs, but also RRs from human serum showed negative correlations with the molecular masses of the cytokines/chemokines recovered and colloid perfusate flow rates (Figure 2 and Figure 3).

### 3.3. Ex Vivo Cytokine and Chemokine Recovery from Human and Porcine Skin

Ex vivo and in vivo experiments were carried out using retrodialysis, where recovery was calculated based on the RL and AL of cytokine or chemokine molecules from the dialysate through the membrane from the inside to the outside of the catheter. Retrodialysis is basically reverse microdialysis. 

**Figure 4:**
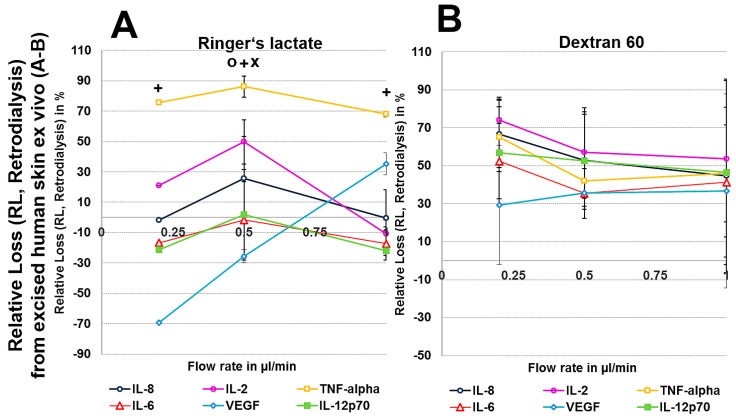

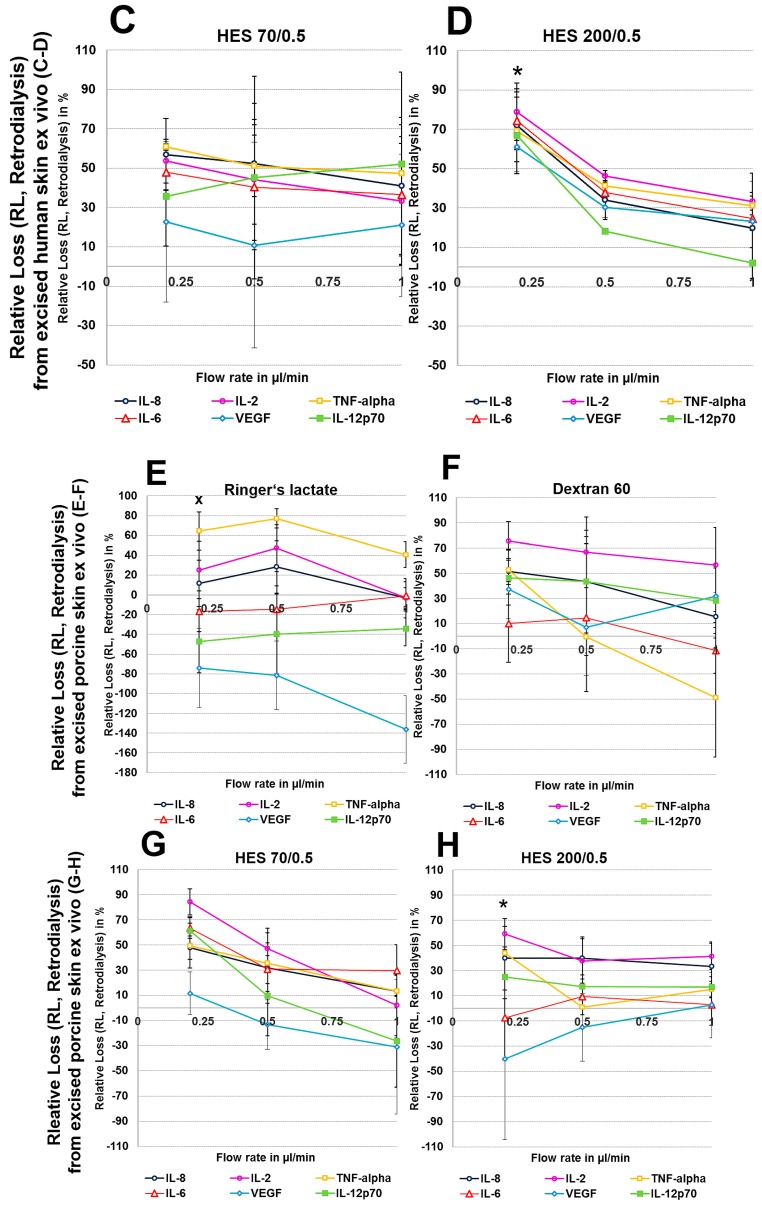
(**A**–**H**): Effect of different colloid perfusates (dextran 60 (**B**,**F**), hydroxyethyl starches (HES) 70/0.5 (**C**,**G**) and 200/0.5 (**D**,**H**) and the crystalloid Ringer’s lactate solution (**A**,**E**)) on relative loss (RL) from excised human (**A**–**D**) and porcine skin (**E**–**H**) during retrodialysis and ex vivo experiments. Each figure represents the mean values with standard deviation of three independent experiments of all six different cytokines at flow rates of 0.2, 0.5 and 1 µL/min. Significance (*p* < 0.05) is indicated by *, x, o, and +. * shows significant differences compared with Ringer’s lactate, x shows significant differences compared with HES 200/0.5, + shows significant differences when compared with HES 70/0.5, and o shows significant differences compared with dextran 60 solution.

**Figure 5:**
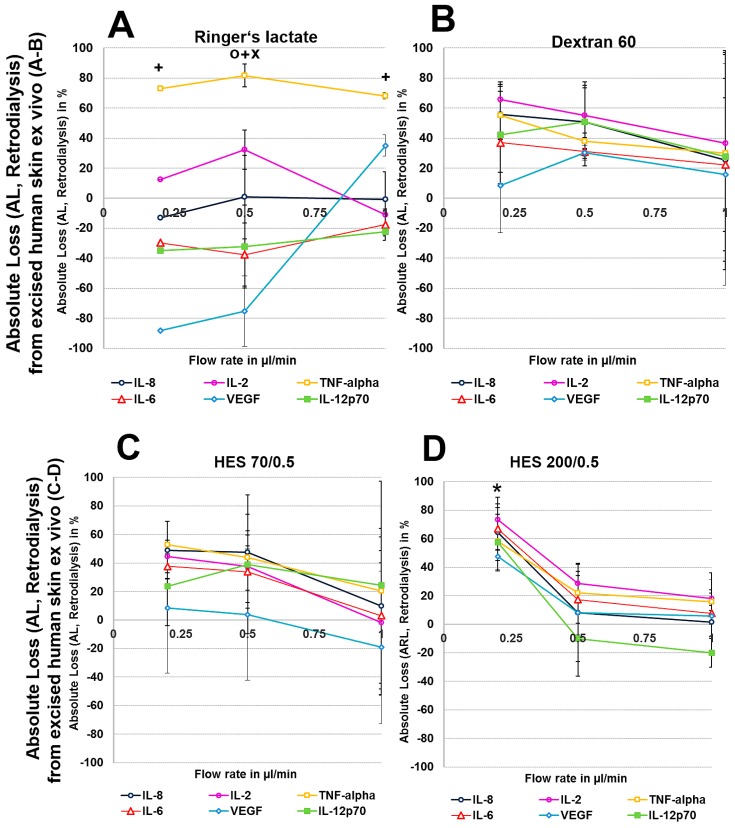

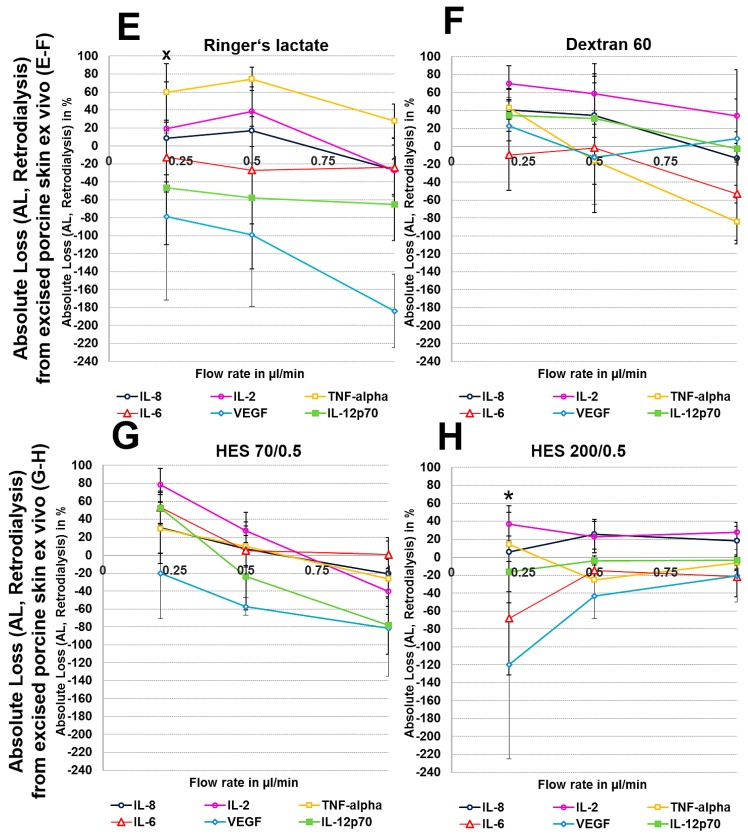
(**A**–**H**): Effect of different colloid perfusates (dextran 60 (**B**,**F**), hydroxyethyl starches (HES) 70/0.5 (**C**,**G**) and 200/0.5 (**D**,**H**) and the crystalloid Ringer’s lactate solution (**A**,**E**)) on absolute loss (AL) from excised human (**A**–**D**) and porcine skin (**E**–**H**) during retrodialysis and ex vivo experiments. Each figure represents the mean values with standard deviation of three independent experiments of all six different cytokines at flow rates of 0.2, 0.5, and 1 µL/min. Significance (*p* < 0.05) is indicated by *, x, o and +. * shows significant differences when compared with Ringer’s lactate, x shows significant differences compared with HES 200/0.5, + shows significant differences compared with HES 70/0.5, and o shows significant differences compared with dextran 60 solution.

To exclude any influence of local human cytokines (porcine cytokines are not detected by the human-specific Cytokine Bead Array) on the retrodialysis experiments in human cadaver skin, we performed conventional microdialysis with all perfusates for 250 min at 0.2 µL/min in three independent experiments after performing basal microdialysis to equilibrate each flow rate for 30 min. We were unable to detect any amount of human IL-2, IL-6, IL-8, IL-12p70, TNF-α, and VEGF in those dialysates. The retrodialysis experiments in porcine or human cadaver skin showed that the dialysate volume increased with increasing flow rate, as expected because the time of exchange with the surrounding tissue was reduced (Table 1). The difference in volume between the Ringer’s lactate and colloid solutions was lower; however, the HES 70/0.5 solution yielded the highest dialysate volumes (Table 1). The volumes obtained from ex vivo experiments using excised human skin were lower than those that were obtained with porcine skin. Using Ringer’s lactate as the perfusate in the ex vivo experiments, the ALs and RLs between cytokines differed, with high values for TNF-α and negative values for the high-molecular-mass molecules IL-12p70 and VEGF, resulting in 2-fold increased VEGF concentrations at flow rates of 0.2 and 0.5 µL/min (Figure 4 and Figure 5). The AL and RLs of all cytokines and chemokines, except for TNF-α, particularly the high-molecular-mass molecules IL-12p70 and VEGF, were strongly increased by colloid perfusates, particularly by the dextran 60 and HES 70/0.5 solutions. The flow rate did not have the same influence on the AL and RL, as that observed in vitro except for with the HES 200/0.5 perfusate (Figure 4 and Figure 5). However, to a lesser extent, the AL and RL were also negatively correlated with the molecular mass and flow rate in the human and porcine skin ex vivo experiments. The AL and RL results that were obtained from porcine and human skin ex vivo experiments using colloids were in a similar range even when higher variations were observed with the different cytokines (Figure 4 and Figure 5). Although HES 200 kDa is almost unable to pass through the microdialysis membrane (the membrane cut-off of 100 kDa excludes 90% of molecules above 100 kDa, according to the manufacturer’s CMA microdialysis), and is therefore able to lower colloid osmotic pressure, this characteristic had an increasing effect on the AL and RL at only 0.2 µL/min in human skin ex vivo, but not for any other flow rate or in porcine skin. Therefore, the inability of HES 200/0.5 with 200 kDa molecules to pass through the membrane appeared to decrease the AL and RL, which might be explained by an intraprobe increase in the colloid osmotic pressure.

### 3.4. In Vivo Cytokine and Chemokine Recovery from Porcine Skin by Retrodialysis

For Ringer’s lactate solution, decreases in dialysate volumes were observed at flow rates of 0.5 and 0.2 µL/min, suggesting tissue loss. In general, the dialysate volume increased with flow rate, which was consistent with the reduced time that was allowed for exchange across the membrane. Among the colloid perfusates, HES 70/0.5 yielded the highest dialysate volumes (Table 1).

However, when colloid perfusates were used, the highest RLs and ALs were achieved at 0.5 µL/min for most cytokines and chemokines, except for VEGF. At this flow rate, HES 70/0.5 and dextran 60 showed higher ALs for IL-2, IL-6, IL-8, TNF-α, and IL-12p70 than Ringer’s lactate, in contrast to the HES 200/0.5 solution (Table 2).

### 3.5. In Vivo Cytokine and Chemokine Recovery from Porcine Skin after Intradermal Injection

To test whether retrodialysis might overrate the recovery in vivo due to adherent effects of the surrounding tissue, we performed conventional microdialysis after injecting the mixture of six recombinant cytokines intradermally around a catheter that was placed into the dermis in the exact same manner, as described previously. The highest RR values were achieved with HES 70/0.5, observing up to 12% recovery for IL-8 and up to 10% recovery for VEGF (Table A2). The RR values achieved using HES 70/0.5 were higher than those achieved with the dextran 60 and HES 200/0.5 solutions. When comparing these results with the retrodialysis data obtained from ex vivo and in vivo experiments (Figure 4 and Figure 5, Table 2), these RR values were lower, except for VEGF (Table A2), but were more comparable to those that were observed in the in vitro experiments with human serum (except for IL-8, Figure 2E–H).

### 3.6. Ex Vivo Cytokine Collection after Skin Trauma (Insertion) and UV Irradiation

When comparing the results of the crystalloid perfusate Ringer’s lactate solution and the colloid perfusates HES 200/0.5 and dextran 60, all of the perfusates were capable of yielding detectable amounts of the cytokines and chemokines in the dialysates, except for IL-2 when using HES 200/0.5 and after skin trauma by catheter insertion (Table 3). Both colloid perfusates were clearly superior in sampling the cytokines IL-6, IL-8, and IL-10 and the chemokines IP-10 and MCP-1 after UVB irradiation and insertion, resulting in a much higher AUC when compared with that achieved with the crystalloid perfusate Ringer’s lactate solution (Table 3). Regarding the cytokine TNF-α, similar to the in vitro experimental results with human serum, no clear differences between the crystalloid and colloid perfusates were observed (Table 3). The sampling of IL-2 resulted in an increased AUC only with dextran 60 after UVB irradiation. Regarding the smallest cytokine (IL-8), dextran 60 showed a notably higher AUC after UVB irradiation and skin trauma (catheter insertion) than HES 200/0.5 (Table 3).

Finally, the lipophilic emulsion Lipvenös^®^, containing soy bean proteins and triglycerides, was unsuitable for sampling cytokines and chemokines due to very low RRs and ARs. First, we were unable to perform microdialysis at a flow rate of 0.2 µL/min, which was expected to show the highest RR or AR. We then used higher flow rates of 0.5, 1, and 5 µL/min. Second, the dialysate volume that was obtained with Lipvenös^®^ was similar to that obtained with Ringer’s lactate solution in vitro, but very low ARs of 5.7% ± 3.7 for the crystalloid solution Ringer’s lactate and 3.3% ± 1.2 for Lipvenös^®^, at 1.0 µL/min both from Ringer’s lactate as the surrounding medium, were observed for only the smallest chemokine, IL-8 (data not shown). The RRs of the remaining cytokines were below 0.35% with Lipvenös^®^ in vitro (data not shown). Therefore, this perfusate appeared to be unsuitable for further investigation. 

## 4. Discussion

Sampling macromolecules, including high-molecular-mass proteins, such as cytokines and chemokines, via microdialysis has gained increasing interest, as it is the only method that allows for the in vivo detection of intercellular signalling molecules without destroying the tissue of interest [17,24]. New array platforms, including flow cytometric-based bead arrays, allow for the simultaneous measurement of multiple analytes in the range of 5 to 5000 pg/mL [21,22,45]. Low-molecular-mass analytes can be sampled at short intervals (minutes), allowing precise reproduction of the in vivo situation. In contrast, low recovery of high-molecular-mass proteins and sampling intervals of up to 8 h have reportedly yielded a sufficient amount of analytes, even when using high cut-off microdialysis membranes of 100 kDa [9,11], which is very unlike the actual in vivo environment. Apart from crystalloids, such as Ringer’s lactate and 0.9% sodium chloride, the most commonly used perfusates for collecting macromolecules via microdialysis are dextran (60 and 70 kDa), bovine and human serum albumin (3.5%, 7% or 10%), heparins and beta-cyclodextrin for lipophilic analytes because they all are somehow capable of preventing dialysate volume loss [19,47,48,49,50,51,52,53]. In 2002, IL-6 was the first cytokine to be sampled from dermal skin using a 100 kDa cut-off membrane, and dialysates were analysed using a commercial Cytokine-ELISA kit [15]. In these experiments, ultrafiltration was problematic because 58% of the dialysate volume was lost when using the perfusate Ringer acetate. This loss was preventable by using dextran 60 as the perfusate. However, even with dextran solutions, the recovery of IL-6 was only 5%. To sample the cytokine TNF-α, even antibody-coated microspheres have been tried in vitro [19], which strongly increased the recovery of TNF-α from 5.5% to 60.4% at a flow rate of 1 µL/min. However, due to ethical concerns, this method is not easily feasible in humans. 

The fact that recovery might be influenced by external conditions, such as temperature and cytokine binding to inlet or outlet tubing or the membrane, has been concerning, but was investigated previously and excluded [54].

In our study, we investigated the RR and AR of six recombinant human cytokines of increasing molecular size using in vitro, ex vivo, and in vivo experiments. Due to their similarities to interstitial fluid and human skin [36,37], we used human serum and porcine skin, respectively, ex vivo and in vivo. Finally, we used excised human skin ex vivo and in vivo to find the most efficient perfusate (crystalloid or colloids) for investigating intercellular signalling in clinical trials. 

Due to known allergic and anaphylactic reactions to dextran solutions [55,56,57], we investigated HES 70/0.5, HES 200/0.5, and a 30% lipid emulsion formulation based on soy bean proteins and triglycerides that could be used to replace dextran solutions. Using HES 200/0.5, 90% of macromolecules >100 kDa, such as HES 200, are not expected to pass through the 100-kDa cut-off microdialysis catheter, as indicated by the manufacturer. Furthermore, another aim of this study was to test RRs and ARs at different flow rates. Because a sampling volume of at least 50 µL is required for cytometric bead arrays to allow the simultaneous analysis of various cytokines and chemokines, short sampling intervals are desirable to more precisely reproduce the in vivo situation.

In vitro experiments showed that recoveries were dependent on low flow rates, with higher RRs and ARs being observed at low flow rates, and on the size of the cytokine, as the smallest cytokine (IL-8) showed the highest recovery and the largest cytokine (IL-12p70) showed the lowest recovery. Furthermore, whereas the significant AR increases that were observed for the HES 70/0.5, HES 200/0.5 and dextran 60 solutions when compared with the crystalloid Ringer’s lactate solution were based on an increase in the dialysate volumes when Ringer’s lactate was used as the surrounding medium, RR increases were also based on the cytokine concentration within the dialysate when human serum served as the surrounding medium. When testing porcine and human skin, the AR and RR of TNF-α was significantly higher with Ringer’s lactate in all of the ex vivo and in vivo experiments, in contrast to the other cytokines. The variability observed was higher when cadaver skin was used when compared with the in vitro experiments. This is most likely because the cadaver skin was collected from different individuals (human and pigs), contributing to the higher variability observed in the ex vivo experiments. 

Retrodialysis appeared to be disadvantageous in static systems, such as excised cadaver skin, leading to increased membrane concentrations for larger cytokines, such as VEGF. Furthermore, tissue adherent effects might further influence the recovery, as observed with TNF-α.

To avoid any of these effects with excised cadaver skin, we performed in vivo experiments. Experiments with both porcine and human skin in vivo showed that the use of colloid perfusates resulted in increased total amounts of cytokines (AR)s and concentrations (RR)s within the dialysates at lower flow rates for most of the cytokines and chemokines. 

In conclusion, our data indicate that the HES 70 kDa and 200 kDa solutions are suitable perfusates for use in clinical practice with an overall superiority of HES 70 kDa, particularly when dextran solutions need to be replaced. Colloid perfusates, such as HES or dextran solutions, are necessary to prevent fluid loss across the membrane, resulting in increased dialysate volumes, and the final cytokine and chemokine concentrations were compared with those that were achieved with crystalloid solutions. The recoveries obtained using the HES 70 kDa and 200 kDa solutions were not clearly superior to those obtained using the dextran solution. However, overall lower flow rates of 0.5 or 0.2 µL/min are recommended for microdialysis in dermal skin due to higher RRs and ARs when sampling cytokines or chemokines.

## Figures and Tables

**Table 1:** Volume of dialysates (µL) recovered (*n* = 3 with standard deviation, in vivo pooled data from two pigs).

Surrounding	Perfusate	Flow Rate (µL/min)		
medium and setting	solution	0.2	0.5	1.0
Ringer’s lactate	Ringer’s lactate	40.1 ± 6.8	39.0 ± 4.2	48.8 ± 1.1
solution	Dextran 60	188.6 ± 8.4 *	110.9 ± 2.0 *	95.3 ± 4.5
in vitro	HES 70/0.5	144.3 ± 10.2 *	81.2 ± 3.7 *	98.9 ± 15.1
	HES 200/0.5	162.2 ± 4.2 *	91.0 ± 1.4 *	81.7 ± 8.2
Human serum	Ringer’s lactate	0.0	68.6 ± 2.6	46.9 ± 2.2
in vitro	Dextran 60	67.6 ± 2.5 *	67.6 ± 5.5	72.6 ± 8.8 *
	HES 70/0.5	88.6 ± 4.0 *	83 ± 3.6 *	77.2 ± 3.2 *
	HES 200/0.5	81.2 ± 1.6 *	58.3 ± 9.6	69.7 ± 0.5 *
Human skin ex vivo	Ringer’s lactate	45.0 ± 0.1	36.4 ± 3.7	49.8 ± 0.2
	Dextran 60	41.2 ± 0.2 *	46.3 ± 3.3	59.0 ± 11.0
	HES 70/0.5	43.0 ± 2.5 *	50.0 ± 1.7	78.0 ± 1.0
	HES 200/0.5	36.0 ± 6 *	40.5 ± 10.5	51.3 ± 10.3
Porcine skin ex vivo	Ringer’s lactate	52.7 ± 13.8	48.0 ± 15.4	60.6 ± 7.6
	Dextran 60	60.9 ± 3.0	58.3 ± 11.9	66.4 ± 14.2
	HES 70/0.5	64.5 ± 18.6	70.7 ± 8.4	73.5 ± 10.7
	HES 200/0.5	76.4 ± 5.6 *	62.7 ± 5.6	63.2 ± 4.0
Porcine skin in vivo	Ringer’s lactate	37.0	42.1	48.4
	Dextran 60	47.0	47.5	50.0
	HES 70/0.5	58.4	64.0	83.0
	HES 200/0.5	49.0	55.8	69.9

Significance versus Ringer’s lactate (*p* < 0.05) is indicated by *.

**Table 2:** Relative loss (left column) and Absolute loss (right column) from retrodialysis in anaesthetised domestic pigs in vivo (pooled data from two pigs). Crystalloid and colloid perfusates were used for the recovery of six cytokines at flow rates of 0.2, 0.5, and 1.0 µL/min.

	Flow Rate	RL/AL (%)					
	(µL/min)	IL-8	IL-2	TNF-α	IL-6	VEGF	IL-12p70
Ringer’s	0.2	89.6/83.3	75.6/66.5	74.1/73.9	57.1/45.8	−40.7/−13.0	67.3/63.9
lactate	0.5	66.0/65.9	59.9/63.1	59.0/63.7	45.4/49.6	−52.6/−8.7	69.5/72.7
	1.0	82.1/82.3	60.9/63.0	54.7/57.1	43.9/45.6	−7.5/−2.5	71.3/73.2
HES	0.2	92.0/90.7	78.0/74.3	73.0/68.5	52.6/44.7	22.3/9.3	68.1/62.8
70/0.5	0.5	97.0/96.2	86.3/82.4	75.7/68.9	65.9/56.3	10.4/−14.7	86.9/83.2
	1.0	71.8/53.2	44.7/8.2	60.9/35.1	56.8/28.3	4.8/−58.0	61.9/36.7
HES	0.2	65.9/52.1	50.9/52.1	49.7/51.8	26.1/28.1	11.9/−10.1	41.3/46.0
200/0.5	0.5	73.5/69.6	57.8/50.9	51.0/42.5	37.8/28.3	40.5/38.7	63.5/55.7
	1.0	52.2/32.8	41.6/16.7	36.6/7.9	21.2/−9.7	−91.2/−137.1	39.7/14.8
Dextran	0.2	81.6/80.4	64.6/62.3	64.9/62.7	47.5/44.2	−3.2/−9.9	40.5/36.7
60	0.5	76.6/75.4	79.2/78.1	76.4/75.1	64.8/62.9	52.6/50.0	60.9/58.7
	1.0	23.9/23.9	61.6/61.6	54.8/54.8	42.4/42.4	39.6/39.6	64.0/64.0

Perfusates were used for the recovery of six cytokines at flow rates of 0.2, 0.5 and 1.0 µL/min.

**Table 3:** Cytokine concentrations plotted as the area under the curve (pg/mL/m^2^) for insertion and UVB irradiation.

Table 3: AUC in pg/mL			
UVB Irradiation	Ringer’s Lactate	HES 200/0.5	Dextran 60
IL-2	74.8	26.2	336.2
IL-6	268.8	51,941.9	21,236.5
IL-8	3364.8	79,3397.0	149,614.7
TNF-α	472.5	385.0	396.9
IL-10	141,88	368.8	221.2
IP-10	1242.4	547,962.2	666,106.2
MCP-1	46,027.9	50,721,379.5	13,294,473.3
**Catheter insertion**			
IL-2	54.9	0	36.2
IL-6	155.7	4377.2	1,736,517.9
IL-8	320.4	44,305.1	107,388.6
TNF-α	92.9	93.5	92.6
IL-10	14.6	35.12	39.86
IP-10	157.3	605.3	9262.2
MCP-1	9085.7	1,387,311.7	7904.4

## Appendix A

**Table A1:** Characteristics of the different crystalloid and colloid perfusates investigated; HES (hydroxyethyl starch).

Perfusate	Density (28 °C, g/mL)	Kinematic Viscosity (28 °C, mm^2^/s)	Dynamic Viscosity (28 °C, mPa·s)	Osmolarity (mosmol/L)
Ringer’s lactate solution	1.002	0.875 ± 0.004	0.877 ± 0.004	279
HES 70/0.5	1.024	1.912 ± 0.003	1.957 ± 0.003	310
HES 200/0.5	1.019	2.477 ± 0.013	2.523 ± 0.014	308
Dextran 60	1.029	3.082 ± 0.013	3.173± 0.013	340

**Table A2:** Relative recovery (RR, %) from microdialysis after the careful dermal injection of 1600 pg/mL around the microdialysis membrane in anaesthetized domestic pigs in vivo. Microdialysis was thereafter performed at a flow rate of 1 µL/min.

	Volume of Dialysate (µL) Recovered	Flow Rate (µL/min)	IL-8	IL-2	TNF-α	IL-6	VEGF	IL-12p70
HES 70/0.5	40	1.0	12.2	7.4	1.7	8.5	10.2	1.7
HES 200/0.5	39	1.0	0.8	0.0	0.6	0.7	5.3	0.2
Dextran 60	39	1.0	8.8	6.0	3.8	3.6	8.1	1.1
